# Sensitive quantification of the HIV-1 reservoir in gut-associated lymphoid tissue

**DOI:** 10.1371/journal.pone.0175899

**Published:** 2017-04-17

**Authors:** Sara Morón-López, Maria C. Puertas, Cristina Gálvez, Jordi Navarro, Anna Carrasco, Maria Esteve, Josep Manyé, Manel Crespo, Maria Salgado, Javier Martinez-Picado

**Affiliations:** 1 AIDS Research Institute IrsiCaixa, Institut d’Investigació en Cièncias de la Salut Germans Trias i Pujol, Universitat Autònoma de Barcelona, Badalona, Spain; 2 Infectious Diseases Department, Hospital Universitari Vall d’Hebron, Barcelona, Spain; 3 Department of Gastroenterology, Hospital Universitari Mutua de Terrassa, University of Barcelona, Research Foundation Mutua de Terrassa, Terrassa, Spain; 4 Centro de Investigación Biomédica en Red de Enfermedades Hepáticas y Digestivas [CIBERehd], Barcelona, Spain; 5 CIBEREHD (Centro de Investigación Biomédica en Red, Enfermedades hepáticas y digestivas), IBD Unit, Germans Trias i Pujol research Institute, Badalona, Spain; 6 Universitat de Vic–Universitat Central de Catalunya, Vic, Spain; 7 Institució Catalana de Recerca i Estudis Avançats, Barcelona, Spain; CEA, FRANCE

## Abstract

**Background:**

The implementation of successful strategies to achieve an HIV cure has become a priority in HIV research. However, the current location and size of HIV reservoirs is still unknown since there are limited tools to evaluate HIV latency in viral sanctuaries such as gut-associated lymphoid tissue (GALT). As reported in the so called “Boston Patients”, despite undetectable levels of proviral HIV-1 DNA in blood and GALT, viral rebound happens in just few months after ART interruption. This fact might imply that current methods are not sensitive enough to detect residual reservoirs. Showing that, it is imperative to improve the detection and quantification of HIV-1 reservoir in tissue samples. Herein, we propose a novel non-enzymatic protocol for purification of Lamina Propria Leukocytes (LPL) from gut biopsies combined to viral HIV DNA (vDNA) quantification by droplet digital PCR (ddPCR) to improve the sensitivity and accuracy of viral reservoir measurements (LPL-vDNA assay).

**Methods:**

Endoscopic ileum biopsies were sampled from 12 HIV-1-infected cART-suppressed subjects. We performed a DTT/EDTA-based treatment for epithelial layer removal followed by non-enzymatic disruption of the tissue to obtain lamina propria cell suspension (LP). CD45^+^ cells were subsequently purified by flow sorting and vDNA was determined by ddPCR.

**Results:**

vDNA quantification levels were significantly higher in purified LPLs (CD45^+^) than in bulk LPs (p<0.01). The levels of vDNA were higher in ileum samples than in concurrent PBMC from the same individuals (p = 0.002). As a result of the increased sensitivity of this purification method, the Poisson 95% confidence intervals of the vDNA quantification data from LPLs were narrower than that from bulk LPs. Of note, vDNA was unambiguously quantified above the detection limit in 100% of LPL samples, while only in 58% of bulk LPs.

**Conclusion:**

We propose an innovative combined protocol for a more sensitive detection of the HIV reservoir in gut-associated viral sanctuaries, which might be used to evaluate any proposed eradication strategy.

## Introduction

There is an emerging interest in developing safe and affordable curative strategies that would eliminate the need for lifelong antiretroviral therapy (ART) in HIV-1 infected patients, improving the health status and reducing the risk of viral transmission to uninfected individuals. In most clinical trials, HIV-1 plasma RNA and cell-associated viral DNA (vDNA), as well as replication-competent reservoir in circulating CD4^+^ T cells, are the primary end-points analyzed to assess the impact of the different curative strategies, as resting memory CD4^+^ T cells represent the largest number of latently infected cells [1]. However, the vast majority of these cells reside in other anatomical sites such as spleen, lymph nodes, gastrointestinal mucosa and respiratory tract, and the cell equilibrium between them is not well established [2]. From them, the population of T cells in gut-associated lymphoid tissue (GALT) stands for more than 60% of the total body lymphocytes [3]. Of note, gastrointestinal lamina propria is a major site of viral replication and CD4^+^ T cell depletion during acute infection [4,5].

Several studies have demonstrated the persistence of HIV-1 infection in gut-associated CD4^+^ memory T cells in long-term suppressed HIV-1-infected patients [6,7]. However, as reported in the so called “Boston Patients”, despite undetectable levels of proviral HIV-1 DNA in blood and GALT tissue, viral rebound can happen in just few months after ART recess [8]. This fact might imply that current methods are not sensitive enough to detect residual reservoirs, so new approaches are needed to better define and quantify HIV-1 reservoirs in tissue samples.

In gut biopsies, vDNA quantification has frequently been done in the bulk from snap-frozen tissue or in cell suspensions, which are obtained in fresh after collagenase digestion and mechanical disruption [9–17]. Albeit vDNA is frequently detected in samples from ART-treated patients by this method, the limit of detection is lowered as a result of the low frequency, about 1%, of CD4^+^ T cells in those samples [10]. Thus, current guidelines recommend a lymphocyte purification step to improve proviral quantification [18]. Still, substantial cell loss and phenotypic alterations might result from the use of enzymatic treatment, thus limiting further functional analysis.

**Fig 1:**
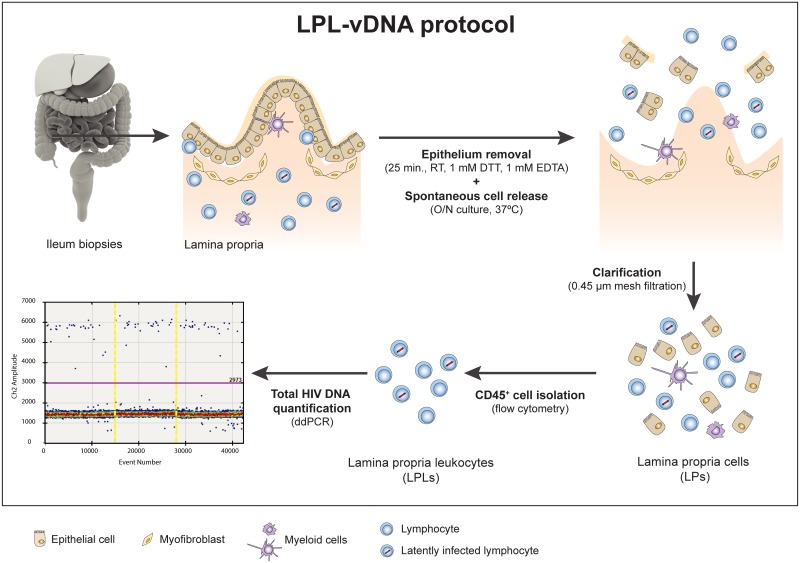
Schematic representation of the LPL-vDNA assay. The cells in blue represent lymphocyte populations and in purple, myeloid populations. LPs: lamina propria cells; LPLs: lamina propria leukocytes; O/N: overnight; ddPCR: droplet digital PCR.

Previous work in HIV negative individuals proved that non-enzimatic methods in GALT tissue cell isolation relay in higher yields of desired cells [19]. Moreover, sorting purification of cell-populations enriched in latently HIV infected cells contributes to increase cell yield and subsequent vDNA quantification [7,20]. Herein, to improve the sensitivity of vDNA quantification from gut associated lymphoid tissue (GALT), we have developed a novel non-enzymatic protocol for purification of lamina propria leukocytes (LPL) from gut biopsies prior vDNA absolute quantification by droplet digital PCR (ddPCR). This new assay (referred now on as LPL-vDNA, Fig 1) renders remarkable cell yield from gut biopsies and significantly increases the sensitivity and robustness of vDNA quantification.

## Methods

### Participants and biopsy collection

We performed a substudy of 12 HIV-infected ART-suppressed individuals belonging to the INDOOR Study (EudraCT number: 2014-004331-39) who were recruited at Vall d’Hebron University Hospital (Barcelona, Spain). We simultaneously collected blood and ileum biopsies from all these individuals. The participants gave their written informed consent, and the institutional review boards from Hospital Vall d’Hebron approved the protocol.

Ileum colonoscopies were performed following a standard procedure. Briefly, subjects underwent a free-fiber diet for 3 days prior the procedure, and had to be in fast condition the day of the colonoscopy. An entire colon exploration until the ileo-caecal valve was performed searching for pathology and obtaining biopsies if needed. Once the ileo-caecal valve was visualized, intubation of 10–15 cm of terminal ileum was performed with the endoscope to obtain four to eight submucosal biopsies that were immediately placed in complete medium: RPMI 1640 with 10% fetal bovine serum (FBS, both from Gibco), supplemented with antibiotics (500 μg/mL pipericillin/tazobactam, Fresenius Kabi) and 1.25 μg/mL amphotericin B (Fungizone, Gibco). Samples were shipped and processed within 1 hour after collection, in order to minimize LPL loss and/or contamination.

### Lamina Propria (LP) cell isolation from ileum biopsies

Biopsies were incubated in HBSS (without Ca^2+^/Mg^2+^, Gibco) containing 1 mM DTT (Sigma) and 1 mM EDTA (Gibco) for 25 min, at room temperature with constant shaking, to remove the epithelial layer. Then, biopsies were transferred again to complete media, containing antifungal and antibiotics, and cultured overnight in 6-well low-binding plates (Costar), at 1–2 biopsies per well. Afterwards, culture supernatants were collected to recover the released cells, and the remaining tissue was disrupted by gentle pipetting. Finally, cell suspensions were cleared from tissue debris using a 40 μm nylon mesh (Falcon).

### LP Leukocyte purification by cell sorting

**Fig 2:**
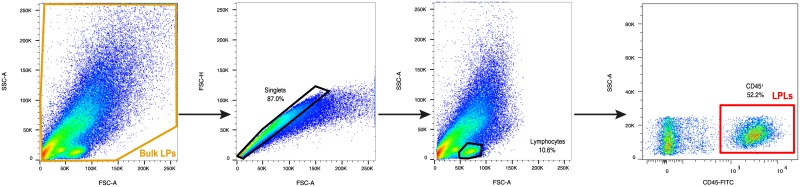
Representation of the gate strategy followed for LPLs isolation. Sorted CD45^+^ cells (LPLs) were framed in the red box.

In order to purify the leukocyte population, lamina propria cells (LPs) were resuspended in PBS (Gibco) containing 2 mM EDTA and 2% FBS, and stained for 30 min at 4°C with the following antibodies: CD45 (FITC), CD3 (APC) and CD8 (PercP) from Becton Dickinson. Sorting of CD45^+^ cells was performed in a BD FACS Aria II Flow cytometer, selecting singlets (FSC-H *vs*. FSC-A) and collecting CD45^+^ from the lymphocytes gate (SSC-A *vs*. FSC-A) (Fig 2). For further calculations, those CD45^+^ cells that were CD3^+^CD8^−^ were assumed to be CD4^+^ T cells, which included those cells that have downregulated CD4 expression because of HIV-1 infection.

Finally, sorted CD45^+^ cells, assumed as lamina propria leukocytes (LPLs), were spun down and resuspended in lysis buffer at a concentration of 5x10^3^ – 5x10^4^ cells/μl (minimum volume of 10 μl). Lysis buffer consisted of 0.1% Triton x-100 (Sigma) and 400 μg/ml Proteinase K (Ambion) in 10 mM Tris-HCl pH = 9.0 (Sigma). Cell lysates were incubated at 55°C for 15–20 h, followed by a 5 minutes inactivation of proteinase at 95°C, prior to storage at –20°C.

### Total vDNA quantification

Lysed extracts from purified LPLs (CD45^+^ cells), bulk LPs and PBMCs from the same participants were simultaneously obtained and used to measure cell-associated HIV-1 DNA by ddPCR. A total of 2–3 μl of cell lysates were mixed with ddPCR supermix and primers/probes (FAM/HEX-ZEN-Iowa BlackFQ double-quenched probes, Integrated DNA Technologies) in a total volume of 20 μl to generate droplets, according to manufacturer’s recommendations. We managed to circumvent potential primer mismatch in individuals’ viral sequence by using two different primers/probe sets annealing to the 5’*LTR* and *GAG* conserved regions of HIV-1 genome [21–23] (Table 1). Here, ddPCR Supermix for Residual DNA quantification was used (186–4037, Bio-Rad). Annealing/extension step was set at 57°C to quantify vDNA using the C1000 Touch^™^ Thermal Cycler (Bio-Rad) and subsequently analyzed using a QX100^™^ droplet reader (Bio-Rad) and the QuantaSoft v.1.6 software (Bio-Rad).

**Table 1:** Primers and probes used for vDNA quantification.

Target	Primer/probe Name	Sequence (5’-3’)	Strand
HIV-1 U5’-LTR	LTR-U5 integrated	GTTCGGGCGCCACTGCTAG	Forward
	LTR-R integrated	TTAAGCCTCAATAAAGCTTGCC	Reverse
	New integrated-2 Probe	CCAGAGTCACACAACAGACGGGCA	Probe
HIV-1 GAG	HIV_F (SCA)	CATGTTTTCAGCATTATCAGAAGGA	Forward
	HIV_R (SCA)	TGCTTGATGTCCCCCCACT	Reverse
	HIV Probe (SCA)	CCACCCCACAAGATTTAAACACCATGCTAA	Probe
*Cellular RPP30* gene	RPP30-F	GATTTGGACCTGCGAGCG	Forward
	RPP30-R	GCGGCTGTCTCCACAAGT	Reverse
	RPP30 Probe	CTGACCTGAAGGCTCT	Probe

PBMCs, bulk LPs and LPLs samples were quantified in duplicate or triplicate, respectively. PBMCs from HIV-negative donors were used as negative controls and assayed in each plate to set the positive/negative threshold for ddPCR analysis, and the number of those negative control wells was the same than replicas for each sample. The RPP30 cellular gene was quantified in parallel to normalize sample input (Table 1) [24].

## Results

### Participants characteristics

The detectability improvement of the novel LPL—vDNA assay (Fig 1) was evaluated in ileum samples from 12 HIV-1-infected individuals. All of them were on suppressive ART composed of two reverse transcriptase inhibitors plus one boosted protease inhibitor. Clinical details of participants included in this substudy are summarized in Table 2. Specifically, at the time of biopsy collection, the median time on suppressive ART was 5 years (interquartile range: 1.3–9.8), and the median CD4^+^ T-cell count was 585 cells/μl (interquartile range: 505–700).

**Table 2:** Participants’ characteristics at baseline.

	n = 12
Age (years), median [IQR]	53 [49–58]
Male, n (%)	8 (67)
Time from HIV-1 diagnosis (years), median [IQR]	21 [17–27]
Time suppressed (years), median [IQR]	5 [1.3–9.8]
CD4^+^ T-cell count (absolute cells/μl), median [IQR]	585 [505–700]

### Cell isolation efficiency of LPL-vDNA assay

After the LPLs isolation protocol, based on a non-enzymatic method, the median number of leukocytes (CD45^+^ cells) recovered was 4.8x10^4^ cells per biopsy (interquartile range, 3.2–6.0x10^4^), which included CD4^+^ T cells (median of 29.4% of CD3^+^CD8^-^ cells in CD45^+^ cells).

### Total proviral DNA quantification comparison in PBMCs, LPLs and bulk LPs

**Fig 3:**
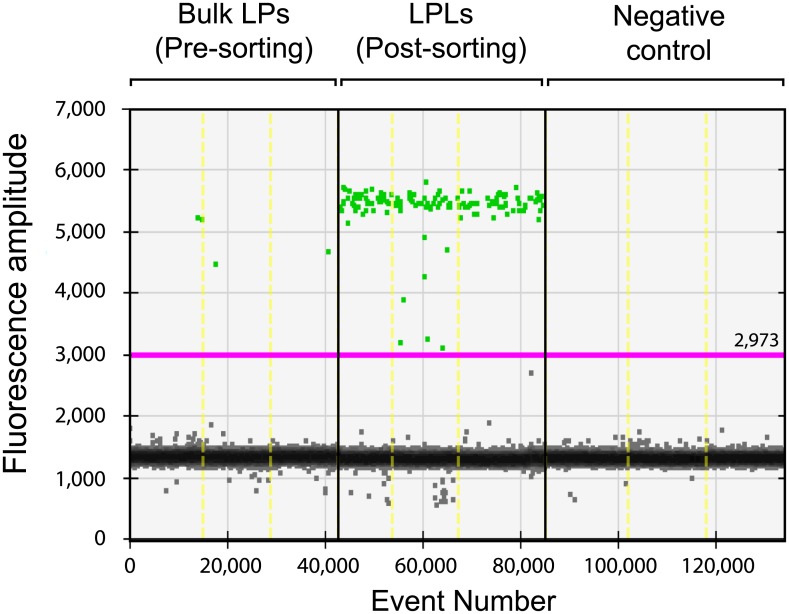
Droplet digital PCR dot plot for *GAG* amplification. Representative results from LPs (pre-sorting), LPLs (post-sorting) and negative controls.

For the absolute quantification of cells harboring proviral DNA, up to 3x10^5^ cells were lysed and directly tested by droplet digital PCR (ddPCR). As shown in Fig 3, vDNA detection by ddPCR was highly improved by LPLs purification by cell sorting of CD45^+^ cells, compared with bulk LPs from the same biopsy.

**Fig 4:**
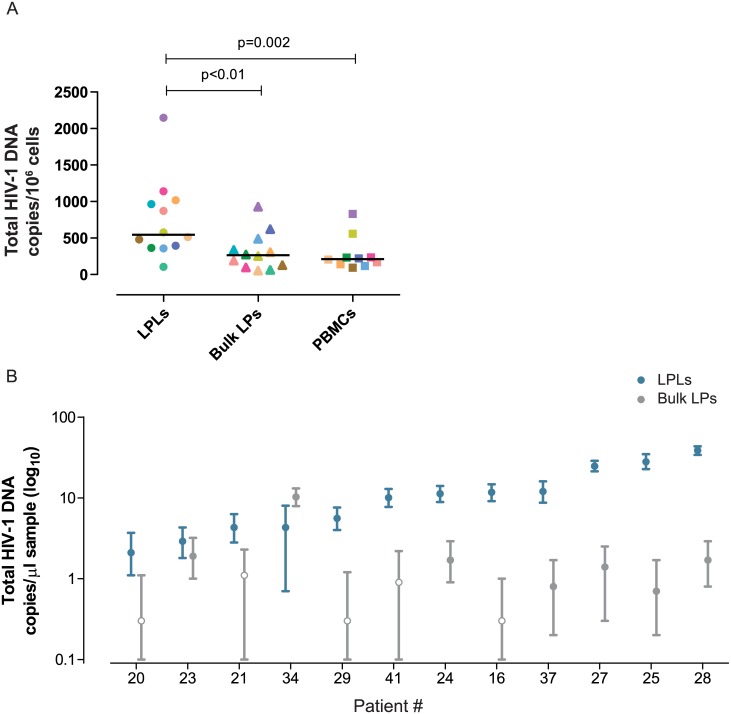
Total HIV-1 DNA quantification by ddPCR. (A) Comparison of total vDNA quantification from LPLs, bulk LPs and PBMCs. (B) Comparison of raw absolute vDNA quantification with Poisson 95% intervals from LPLs and bulk LPs. Clear dots represent the quantifications with the minimum Poisson 95% interval under the limit of detection. Statistically significant p values are showed (Wilcoxon signed rank test).

Despite inter-individual variability, the LPL-vDNA quantification levels were significantly higher in the purified leukocyte population (CD45^+^) than in the unsorted cell suspension (p<0.01, Fig 4A). As expected, the levels of vDNA were higher in ileum samples than in peripheral blood mononuclear cells from the same participants (PBMCs, p = 0.002, Fig 4A)

As a result of the increased sensitivity of this purification method, the Poisson 95% confidence intervals of the vDNA quantification data from LPLs (CD45^+^) were narrower than that from bulk LPs (Fig 4B). Of note, vDNA was unambiguously quantified above the detection limit in 100% of LPL-purified samples, while only in 58% of bulk LP samples.

## Discussion

In this study, we aimed to enhance sensitivity and robustness of vDNA quantification from gut biopsies. As lamina propria leukocytes (LPLs) are mainly CD4+ T cells in healthy individuals, and it is a preferential site for HIV-1 replication, we first set up a novel protocol for LPLs recovery from ileum samples. As described by Mahida et al. [25], epithelial layer removal with DTT prior to 16-hour biopsy culture, allows for spontaneous cellular release from the tissue, thus facilitating disruption of the tissue in absence of enzymatic methods. This method has previously shown to result in greater LPLs yield and purity than enzymatic tissue dissociation, either by low-dose or high-dose collagenase treatment [19]. Further modification of the original protocol by the addition of piperacillin-tazobactam to the collection and culture media prevented microbial contamination from intestinal flora in our tissue cultures by 95% [26]. Minimal cell loss or contamination occurred if samples were shipped just after biopsy collection or after epithelial removal (data not shown). Consequently, this protocol overcomes the need of specific equipment and trained staff at the site of specimen collection. Thus, LPLs-vDNA assay enables multicenter recruitment and sample collection, while systematic processing of tissue biopsies may be done in a reference laboratory [27].

To increase the sensitivity of vDNA detection, LPLs were further purified by flow-based sorting of CD45^+^ cells. This way, not only resting CD4^+^ T cells were tested for proviral content, but also potential productively infected T cells with down-regulated CD4 expression. In addition, ddPCR has been previously demonstrated to be superior to qPCR, in terms of accuracy and precision, for vDNA quantification in PBMCs [28]. In the LPL-vDNA protocol, the advanced ddPCR technology is used for the first time to get absolute vDNA quantification in gut leukocytes, thus enabling the opportunity to accurately evaluate the dynamics of gut HIV-1 levels in interventional studies, as well as to detect low-level reservoir in cure strategies or cohorts of natural or post-treatment controllers.

On consequence, we have developed an innovative combined protocol for a more sensitive detection of the residual HIV infection in gut-associated viral sanctuaries. The combination of epithelial removal and spontaneous cellular release from the tissue resulted in good recovery of lamina propria leukocytes. Viral DNA determinations in the purified LPLs population *versus* the bulk lamina propria cell sample evidenced that the more purified is the sample; the greater is the sensitivity in the detection of vDNA. Although the protocol has been implemented in ileum biopsies, similar performance is expected with colon or rectum biopsies. Thus the combination of the LPL isolation protocol with the more sensitive ddPCR method would allow to accurately detect residual HIV infection in gut-associated viral sanctuaries. Despite we are not measuring replication competent viruses, more sensitive molecular techniques were needed since previous case reports showed HIV viral rebound despite negative detection of HIV-DNA in gut biopsies [8]. This point makes this protocol relevant in the field, as previous data on gut viral reservoirs in clinical studies had been obtained from total tissue [8,9,11–16,29–31]. Thus, the LPL-vDNA technique might become a useful tool to understand the virological outcome of any proposed eradication strategy and to make decisions about treatment interruption.
